# Three-dimensional core-shell alginate microsphere for cancer hypoxia simulation *in vitro*

**DOI:** 10.3389/fbioe.2023.1174206

**Published:** 2023-04-11

**Authors:** Yejiao Ruan, Lingyun He, Jiamin Chen, Jinfeng Wang, Shujing Zhao, Xiaoling Guo, Yao Xie, Zhenzhai Cai, Xian Shen, Chao Li

**Affiliations:** ^1^ The Second Affiliated Hospital and Yuying Children’s Hospital, Wenzhou Medical University, Wenzhou, China; ^2^ Beijing Automation Control Equipment Institute, Beijing, China; ^3^ The First Affiliated Hospital, Wenzhou Medical University, Wenzhou, China

**Keywords:** hypoxia, hypoxia inducible factor (HIF), three-dimensional culture, alginate, tumor microenvironment (TME)

## Abstract

Hypoxia is one of the major causes of cancer resistance and metastasis. Currently, it is still lack of convenient ways to simulate the *in vivo* hypoxic tumor microenvironment (TME) under normoxia *in vitro*. In this study, based on multi-polymerized alginate, we established a three-dimensional culture system with a core-shell structure (3d-ACS), which prevents oxygen diffusion to a certain extent, thereby simulating the hypoxic TME *in vivo*. The cell activity, hypoxia inducible factor (HIF) expression, drug resistance, and the related gene and protein changes of the gastric cancer (GC) cells were investigated *in vitro* and *in vivo*. The results demonstrated that the GC cells formed organoid-like structures in the 3d-ACS and manifested more aggressive growth and decreased drug responses. Our study provides an accessible hypoxia platform in the laboratory with moderate configuration and it may be applied in studies of the hypoxia-induced drug resistances and other preclinical fields.

## 1 Introduction

Gastric cancer (GC) is the third leading cause of malignancy-related deaths worldwide, especially for East Asia, its morbidity and mortality rank among the top five cancers (Ferlay et al., 2021). Due to the difficulties of early diagnosis, most patients with GC are in stage IV at the time of diagnosis, missing the optimal opportunity for surgery. Chemotherapy and radiotherapy are the major curative options for these patients. However, it was found that many patients often developed resistance and eventually passed away from remote metastasis of GC (Ajani et al., 2017).

It is well known that the tumor microenvironment (TME) plays a vital role in tumor malignancy, resistance, and metastasis (Gao et al., 2016, K. G. K; Deepak et al., 2020). Hypoxia is the universal characteristic of TME in all solid tumors including GC (Schito and Rey, 2017; Depping et al., 2019). By genetic recombination or mutation (Semenza, 2014; Pei et al., 2021), cancer cells often acquire more aggressive phenotypes, such as proliferation, migration, invasion, and metastasis in the hypoxic state (He et al., 2017). Hypoxia also has huge adverse impacts on the permeability of chemotherapeutics resulting in ineffective clinical trials (Chambers et al., 2014; Lee et al., 2018). Hypoxia-inducible factor-1 (HIF-1), represented by HIF-1α, performs critical roles in the cell response to hypoxia (Papandreou et al., 2006; Xia et al., 2020). It involves angiogenesis, the energy metabolism pathway reprogramming, induced immune surveillance escape, etc., (Sumiyoshi et al., 2006), and is closely related to chemotherapy resistance (Zhou et al., 2018). For a long time, hypoxia and HIF-1α in TME are considered to be the main factors affecting malignant progression and patient survival. But the underlying mechanism remains to be further revealed.

The traditional two-dimensional cell culture system (2d) has difficulty in accurately replicating the subtle relationships of multiple factors in TME, which frequently leads to the frustrating discrepancy between excellent results *in vitro* and weak effectiveness or fully failed application *in vivo* (Walsh et al., 2014). In addition, these experiments are usually expensive, labor-intensive, and most of the experiments failed in the final stage of the clinical trial. Especially in the R&D of anticancer drugs, history showed that less than 10% of candidates of potential anticancer drugs were commercialized (Knobloch and Rüther, 2008). One of the fundamental reasons is the 2d culture can hardly reflect the TME hypoxia *in vivo* (Whitman et al., 2019; Alizadeh et al., 2020).

Compared with 2d culture, a variety of TME parameters can be reproduced in 3d culture systems (O'Connor et al., 2015). Many results have shown higher chemical tolerance for cancer cells in 3d culture *in vitro* (Rodríguez et al., 2018), and well coincide with the clinical prognosis of the patients (Walsh et al., 2014; Read et al., 2018). At present, most 3d culture is based on commercial products (such as Matrigel, AlgiMatrixTM, etc.), Generally, these products are expensive and difficult to simulate the hypoxia state of TME *in vivo*. Therefore, it is urgent to develop an economical and practical method for oxygen diffusion barrier, to establish *in vitro* 3d culture models that can simultaneously reproduce the physiological and/or pathological characteristics of the TME *in vivo* (Benien and Swami, 2014; Calpe and Kovacs, 2020).

Alginate is a linear anionic polymer isolated from marine brown algae. The alginate-based polyporous hydrogel can be easily produced by cross-linking the carboxyl groups with divalent cations. Alginate-based hydrogel is inert and many advantages have been revealed such as being stable at room temperature, non-toxic, easy to sol-gel, and it has no concerns about the animal origin products. Therefore, it has been widely used in the food industry and biomedicine (Chandrasekaran et al., 2006). Moreover, different from other 3d models, alginate-based droplets are stable, uniform, and can be manipulated independently (Aggarwal et al., 2020; Xia et al., 2020).

Although alginate-based hydrogel is considered to be a multifunctional system that can be applied to different types of cells (Knobloch and Rüther, 2008; Benien and Swami, 2014), it is still lacking a simple, highly reproducible hypoxia 3d culture system. Herein, we establish an alginate-based 3d culture system with a core-shell structure (3d-ACS) and construct an effective oxygen diffusion barrier to simulate GC hypoxia *in vivo*. By comparison between 3d-ACS, 2d culture, and the animal model, the influences of 3d-ACS on drug response and the changes in GC cell malignancy have been investigated systemically. The results demonstrated that the easily prepared 3d-ACS can be used for 3d culture of GC cells and create a mimetic hypoxic TME inside the core-shell structure. The 3d-ACS-cultured GC cells manifested aggregated growth and showed more malignant characteristics, including stronger invasion and migration, as well as significant resistance to the first-line chemotherapeutics. Our results demonstrated that the 3d-ACS faithfully recapitulated hypoxia of TME *in vivo*. Our 3d-ACS system established a valuable tool to shorten the discrepancy between 2d culture and individual patient trials.

## 2 Materials and methods

### 2.1 Materials

B27, EGF, FGF, DMEM medium, fetal bovine serum (FBS), penicillin and streptomycin (100×), Matrigel, Presto blue cell viability reagent, Calcein-AM, and transwell were bought from BD Biosciences (Mountain View, CA, United States). Sodium alginate, barium chloride, calcium chloride, sodium citrate, sodium chloride, EDTA, paraformaldehyde, [Ru (dpp)_3_]^2+^ Cl_2_ and crystal violet were obtained from Aladdin Co., Ltd. (Shanghai, China). Gastrin, Annexin V, propidium iodide (PI), 5-FU, and cisplatin were purchased from Sigma-Aldrich Co., Ltd. (St. Louis, MO, United States). Antifade mounting buffer, 4′,6-diamidino-2-phenylindole (DAPI), 3,3′-diaminobenzidine (DAB), and H&E staining kit, were bought from Beyotime Co., Ltd. (Beyotime, Shanghai, China). cDNA Reverse Transcription Kits were obtained from TaKaRa Bio (Takara, Dalian, China). Gastric cancer cell lines of MGC-803, AGS, SGC-7901, and BGC-823 were obtained from the National Collection of Authenticated Cell Cultures (NCACC, China). The vendor and products number of the primary and second antibodies were listed in Supplementary Table S1 (Supplementary Material).

### 2.2 Preparation of alginate-based 3d microsphere culture system with core-shell structure (3d-ACS)

Various quantities of sodium alginate were weighed and dissolved in DMEM under an ice bath to construct a series of alginate solutions with concentrations of 0.5%, 0.6%, 0.7%, 0.8%, and 0.9%. The solutions were sterilized under 254 nm UV light for 30 min, and 10% FBS, and 1% penicillin/streptomycin were supplemented. Then, the MGC-803 cells were added and mixed evenly (1 × 10^5^ cells/mL).

The cell suspension was drawn into a syringe with a needle of 27 G and slowly dropped into the calcium chloride saline solution (100 mM) by an auto-pump. The droplets (3 days-AC, without shell) were solidified for 10 min, washed 3 times, transferred to a 96-well plate (2-3 microspheres/well), and cultured in medium supplemented with B27 1×, FGF 200 ng/mL, EGF 50 ng/mL, Gastrin 1 nM. At the set time, a few fields of view were randomly selected and recorded under the microscope.

After being cultured for 7 days, the microspheres were immersed in fresh 0.5%, 0.6%, 0.8%, and 0.9% alginate solution (prepared as mentioned above) for 5 min. In the same way, the shell structures were constructed by polymerization of a new layer of alginate hydrogel. The fabricated 3d-ACS was cultured in a new 96-well plate, and relative parameters were recorded as routine.

### 2.3 Cell release from 3d-ACS

The dissolving buffer was prepared as the previous report (Akeda et al., 2009) (0.15 M NaCl, 30 mM EDTA, 55 mM sodium citrate). The 3d-ACS were collected and mixed with dissolving buffer (1:10–20, v/v). With regular gentle inversion for 10 min at 4°C, the 3d-ACS was disaggregated completely. The mixture was centrifuged at 200 *g* for 5 min to collect the released cells, and the cells were counted by a hemocytometer with trypan blue staining (0.4% in PBS) for later experiments.

### 2.4 The characteristic of the cell in 3d-ACS

Cell viability: Cell viability in the 3d-ACS was analyzed using the Presto Blue Cell Viability Reagent. On the 7th day of culture, after the 5 microspheres of 3d-ACS were gently re-despatched and covered with cell viability reagent (final 1×), the 3d-ACS were incubated at 37°C for 3 h, and the fluorescence was detected in a microtiter plate (ELISA) reader (Thermo Multiskan MK3, United States) at ex/em: 560/590 nm.

Membrane integrity: Several 3d-ACS were collected and washed with saline to remove the residual medium and debris. An appropriate volume of Calcein-AM was added (2 μmol/L) and incubated at 37°C for 30 min. Subsequently, the PI solution was replenished (3 μmol/L) for 10 min. After being washed 3 times with saline, the microspheres were mounted in a stainless steel circle filled with an antifade mounting buffer on the glass slide. The fluorescence was observed under a confocal microscope (Leica TCS sp8) to evaluate the viability of MGC-803 cells in the 3d-ACS (Calcein-AM, ex/em: 488/520–600 nm. PI, ex/em: 561/630–700 nm).

### 2.5 Sectioning, H&E, immunohistochemistry (IHC), and immunofluorescence staining of 3d-ACS

At the scheduled time, the 3d-ACS was taken out from the well and solidified with 100 mM barium chloride for 10 min. After washing, the 3d-ACS was fixed overnight in 4% paraformaldehyde at 4°C. The fixed 3d-ACS was dehydrated, embedded, and sectioned (4 μm) as routine, and the slide was subjected to the H&E staining. In addition, the sections were also deparaffinized and blocked as routine for IHC and immunofluorescence staining (detailed in Supplementary Material S1). The images of H&E, IHC, and fluorescence signals were captured under microscopy (Leica DM2500). The IHC quantitative analyses were conducted on the basis of 3 high-power fields of images. The integrated option density (IOD) and positive area were obtained by the FiJi software (http://fiji.sc/), and the score of expression was calculated as formula (1).

Mean density=IOD/area (1)

### 2.6 Intracellular hypoxia evaluation in 3d-ACS

The MGC-803 cells were plated at a density of 1 × 10^5^/mL and 10 μg/mL of [Ru (dpp)_3_]^2+^ Cl_2_ (hypoxia-specific probe) was supplemented. The cells were cultured in different oxygen chambers (O_2_: 1%, 5%, 10%, and 15%) and normoxia (20% O_2_) with 5% CO_2_ for 24 h, and the [Ru (dpp)_3_]^2+^ Cl_2_ signals were reordered under the microscope (Leica DM2500). The fluorescence variation of [Ru (dpp)_3_]^2+^ Cl_2_ was analyzed by Fiji software and the curve between the mean intensity of [Ru (dpp)_3_]^2+^ Cl_2_ and hypoxia was plotted. To monitor the intracellular hypoxia of 3d-ACS and compare the induced hypoxia, the probe of [Ru (dpp)_3_]^2+^ Cl_2_ (10 μg/mL) was added to 7 days cultured 3d-ACS, 3d-AC (without shell) and Matrigel (in normoxia). After 24 h extra culture, the intracellular fluorescence intensity of [Ru (dpp)_3_]^2+^ Cl_2_ and the oxygen concentration in the 3d-ACS, 3d-AC, and Matrigel were evaluated according to the curve mentioned above.

### 2.7 Cytology tests of the cell in 3d-ACS

Wound healing assay: The GC cells (take MGC-803 cell as the representative) were released from the 3d-ACS and resuspended in 6-well plates at a density of 1 × 10^5^/mL. A linear wound was scrapped by a nonopening Pasteur pipette across the confluent cell layer. Cells were washed 3 times and the sizes of wounds were observed and measured at the indicated times.

Migration: In the same way, the MGC-803 cells were released from 3d-ACS and resuspended in DMEM (serum-free) at a density of 1 × 10^5^/mL. The cell suspension was added to the up chamber and the complete medium (10% FBS) was filled in the bottom chamber. After the chambers were cultured in normoxia for 12 h or 24 h, the up chamber was taken out and the cells in it were fixed and stained with crystal violet (0.1%). The cells in the bottom side of the chamber were photographed under a microscope (Nikon Ti). The crystal violet-positive cells were counted by Fiji software.

Invasion: The stock solution of Matrigel was diluted at 1: 20, and placed in the up chamber of the Transwell. The rest steps were identical to the experiment of migration.

### 2.8 Drug responsiveness

After 14 days culture, the GC cells (MGC-803, AGS, SGC-7901, and BGC-823) in the 3d-ACS were treated with a series concentration of 5-FU or cisplatin. Then, the Presto Blue Cell Viability Reagent was added to each well (containing 5 microspheres). The cell viability test was conducted as mentioned above, and the half-maximal inhibitory concentrations (IC50) were calculated by Graphpad prism 6 (https://www.graphpad.com/).

### 2.9 Molecular biology experiments of hypoxia-induced mutation

Western blot: For protein extraction, the GC cells in 3d-ACS were released and lysed in 50–100 μL RIPA buffer and 20 μg of proteins was used for SDS separation. Antibody incubation of blots was performed according to standard protocols. The following antibodies were used: p53, PHD2, MMP2, HIF-1α, MRP1, and GAPDH, and the secondary antibody (detailed in Supplementary Table S1).

Quantitative RT-PCR: In order to further compare the mRNA transcription levels of 3d-ACS and 2d cultured GC cells, the total RNA in the released cells was extracted using Trizol reagent, and transcribed to cDNA with the cDNA Reverse Transcription Kit. The housekeeping gene (GAPDH) primers were added as an internal control. The mRNA expression was analyzed *via* quantitative RT-PCR using SYBR Green (Bio-Rad, Hercules, CA). The sequences of the primer for relative genotype variation were listed in Supplementary Table S2 (Supplementary Materials), and the relative expression was determined by the 2^−ΔΔCt^ method, and each gene was normalized against to GAPDH level.

### 2.10 Xenograft tumor model

The Balb/c-nude mice (male, 6 weeks old, 20–25 g) were purchased from Shanghai Slac Laboratory Animal Co., Ltd. (Shanghai, China). All mice were maintained in the animal house facility with free access to food and water. All procedures for animal experiments were conducted with the guideline of the Ethics Committee for Animal Experimentation of Wenzhou Medical University (SYXK-2020-0014). To establish the xenografts model, 5 microspheres of 3d-ACS culture (with GC cells ∼1.0 × 10^5^) were subcutaneously inoculated into the posterior flank of the mice and the 2d cultured cells (1.0 × 10^5^) as control. The tumor sizes were measured with a caliper and volume was calculated according to Eq. 2:

$$Volume=\left(length\times {width}^{2}/2\right)$$ (2)

After 3 weeks, the mice were sacrificed and the tumors were dissected for weight assessment, H&E staining, and other experiments.

### 2.11 Statistical analyses

The values were presented as the mean ± standard deviation (SD), and all the data were obtained from at least three independent studies. The statistical analyses were determined by one-way ANOVA test or paired *t*-test by GraphPad Prism (v6, GraphPad Software Inc., CA, United States). IC50 was calculated with non-linear regression, and the survival rates were analyzed by log-rank test. The difference thresholds were set at *p*-value less 0.05 and *p*-value less 0.01.

## 3 Results

### 3.1 Proliferation of GC cells in alginate-based microspheres (core)

In order to effectively evaluate the gel formability of alginate and the growth of GC cells, a series of alginate solutions were introduced to prepare the core microspheres. The results showed that the alginate microsphere was very fragile if the concentration was below 0.6%, making it difficult to operate in the later experiments. On the contrary, the cell was hardly proliferating in conditions of alginate concentration higher than 0.9% (data not shown). Therefore, the 0.6∼0.9% concentrations of alginate were chosen for the later studies.

**FIGURE 1:**
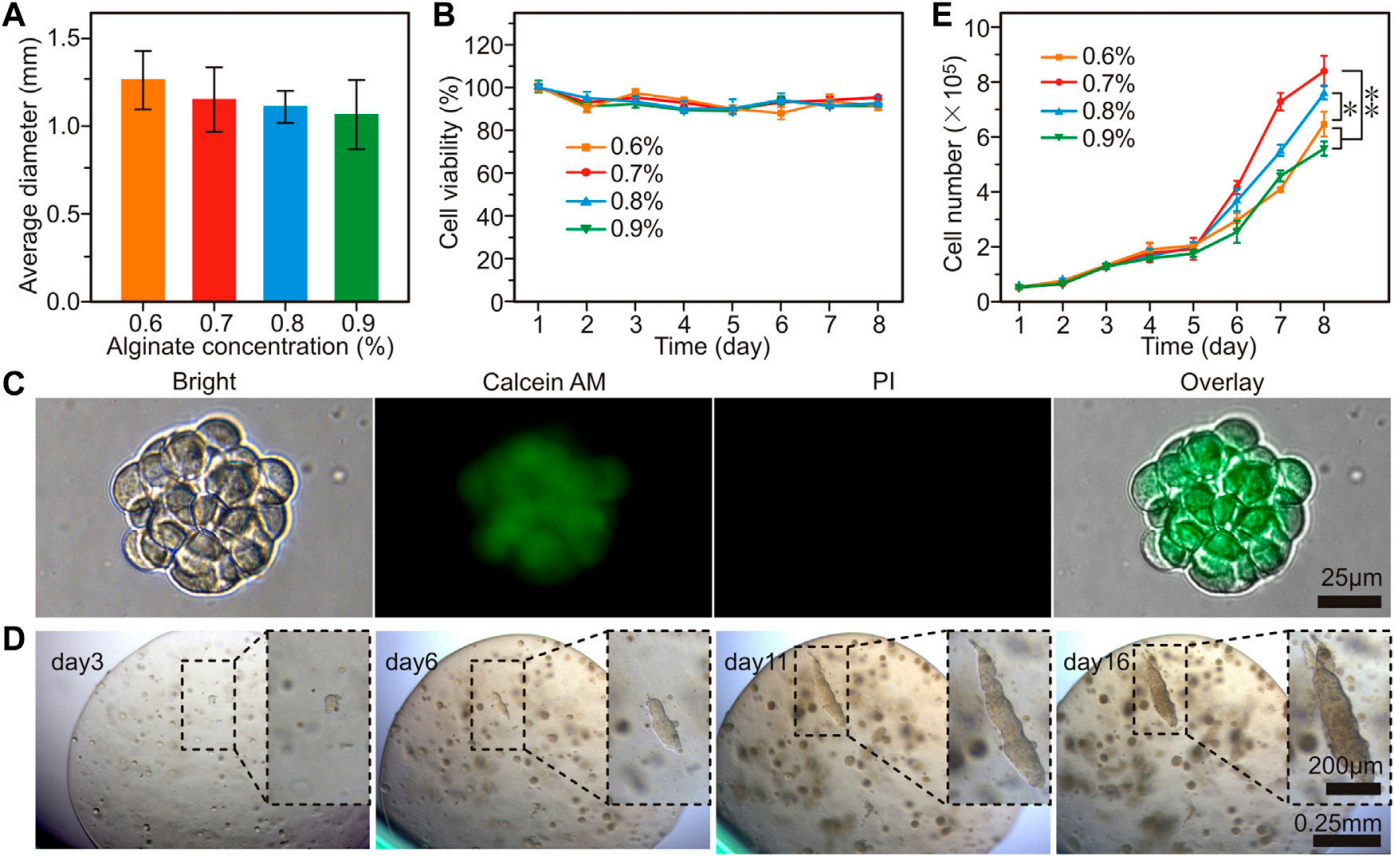
The proliferation of GC cells (MGC-803 cell as the representative) in alginate-based microspheres. **(A)** The average diameter of microspheres prepared with different concentrations of alginate. **(B)** Cell viability of the MGC-803 cells cultured in alginate microsphere. **(C)** Calcein-AM/PI staining of GC cell clusters released from alginate microspheres. **(D)** Dynamic morphology of MGC-803 cell clusters proliferated in 0.7% alginate microspheres. **(E)** Cell counting in different concentrations of alginate microspheres. *n* = 5 per group, *: *p* < 0.05, **: *p* < 0.01.

In natural gravity conditions, the metrology test found that the microspheres with average diameters of 1.25, 1.23, 1.20, and 1.18 mm can be obtained from alginate solution with concentrations at 0.6, 0.7, 0.8, and 0.9% respectively (Figure 1A, Supplementary Figure S1A). Since it needs some time for the 2d educated cells to adapt to the 3d environment, in the first 5 days, the cell viability decreased slightly and the cells were basically maintained in the lag phase. From the 6th day, the trypan blue staining and cell counting revealed that the cell viabilities were higher than 90% (Figure 1B). The Calcein-AM/PI staining indicated that the GC cells maintained acceptable viability in these microspheres (Figure 1C). The GC cells multiplied in the microspheres and gradually developed cell clusters as the culture time went on (Figure 1D). On ∼22nd day, the clone closed to the surface and began to squeeze out due to the limited space and the cavity formed on the edge of the microspheres, and the metastasis-like structure configured along the direction of the cavity (Supplementary Figure S1B, C). The cell proliferation curve demonstrated that the GC cells began to proliferate quickly after the 5 days adaptive phase and maintained the fastest proliferation in 0.7% alginate (*p* < 0.01, Figure 1E). According to the cell viability and proliferation curve, the 0.7% alginate was chosen to form the microspheres.

### 3.2 Activities of MGC-803 cells in 3d-ACS

**FIGURE 2:**
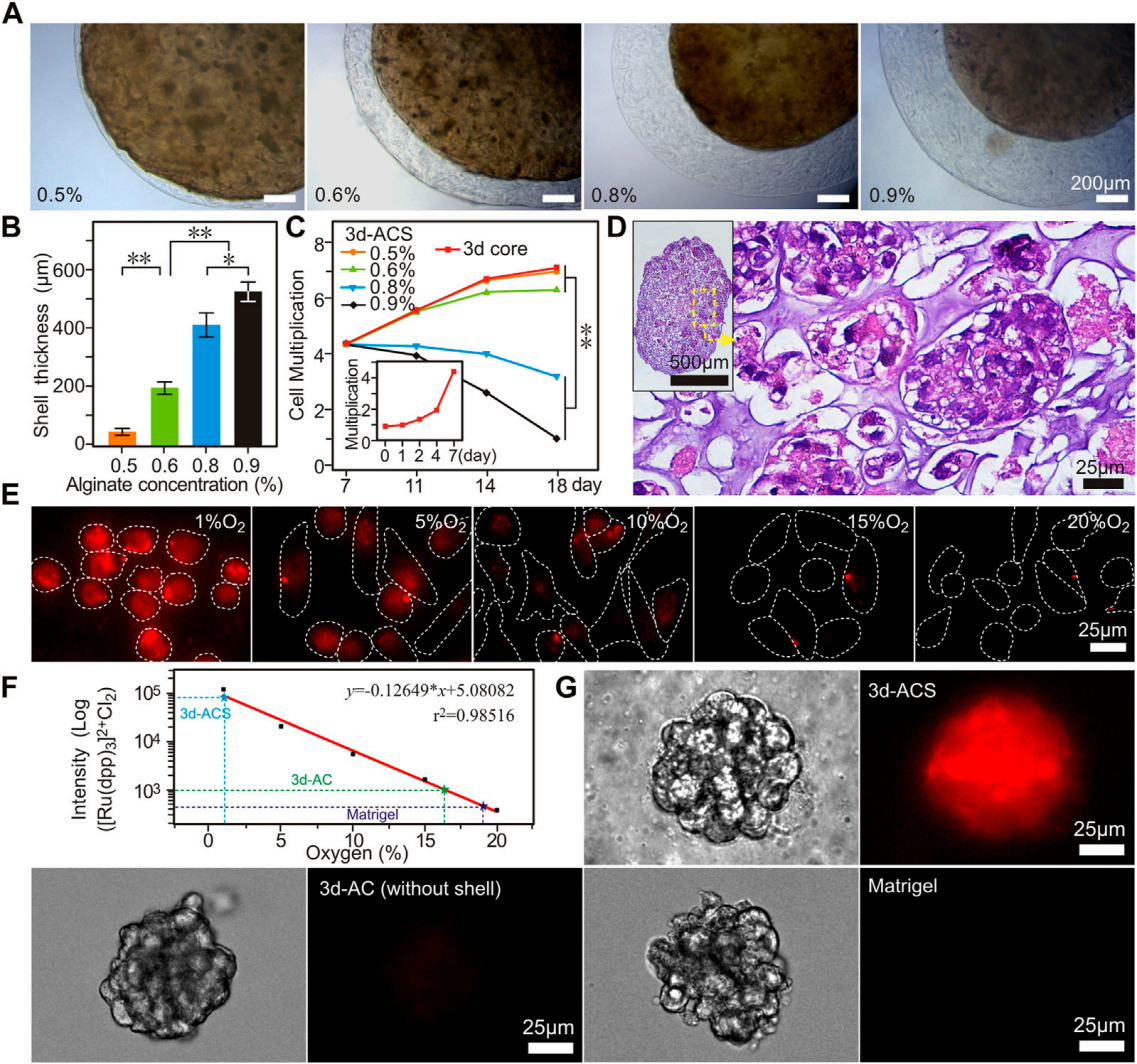
The core-shell structures (3d-ACS) with GC cells that constructed with 0.7% alginate (core) and 0.6%–0.9% alginate (shell). **(A)** The shell structure is formed by polymerization with different alginate solutions. **(B,C)** Quantified thickness of the shell structures and cell proliferation in 3d-ACS (Inset C, cell proliferation in 1–7 days before shell structure formation). **(D)** H&E staining of the 3 d-ACS, the cancer cells grew in 3d-ACS and the organoid-like structure built up gradually. **(E)** Fluorescence of [Ru (dpp)_3_] _2_+ Cl2 in GC cells cultured (2d) in hypoxia (1, 5, 10, 15% O_2_), normoxia (20% O_2_). **(F)** Linear relationship between [Ru (dpp)_3_] _2_+ Cl2 intensity and O_2_ concentration. **(G)** Cell mass fluorescences cultured in 3d-ACS, 3d-AC (without shell), and Matrigel in normoxia (20% O_2_). *n* = 3 per group, **: *p* < 0.01.

The hydrogel microspheres provided a 3d culture matrix for GC cells, but it was insufficient to establish the oxygen diffusion barrier. In order to survive in the core, the cancer cells gradually molded and assembled a solid entity with higher stiffness through physical effects such as extrusion and penetration (Lu et al., 2019). While at the edge, due to the network of collagen, vascular and other matrices, the stiffness in this region is usually slightly lower than that of the core site (Yang et al., 2018). Therefore, after the GC cell grew in 0.7% alginate cores and clones formed (∼7th day), an additional shell structure was polymerized with different concentrations of alginate (0.5 ∼ 0.9%) on the surface of the initial cores to construct the oxygen diffusion barrier, namely, 3d-ACS (Figures 2A, B).

Considering the factors of the uneven stiffness of real solid tumors, and formability, viscosity, and shell thickness of alginate, the 0.6% alginate was chosen to construct a softer shell for the following research (Figure 2C), and the prepared 3d-ACS was cultured in normoxia conditions (20% O_2_, 5% CO_2_). The cell number, activity, and other parameters demonstrated that the 3d-ACS severely impacted cell proliferation (Figure 2C). After additional 11 days of culture in normoxia, the H&E staining showed that the 3d-ACS was brimmed with granules of clones, and the cancer cells were arranged regularly, and the organoid-like structure formed gradually (Figure 2D).

### 3.3 Induced hypoxia in 3d-ACS under the normoxia

**FIGURE 3:**
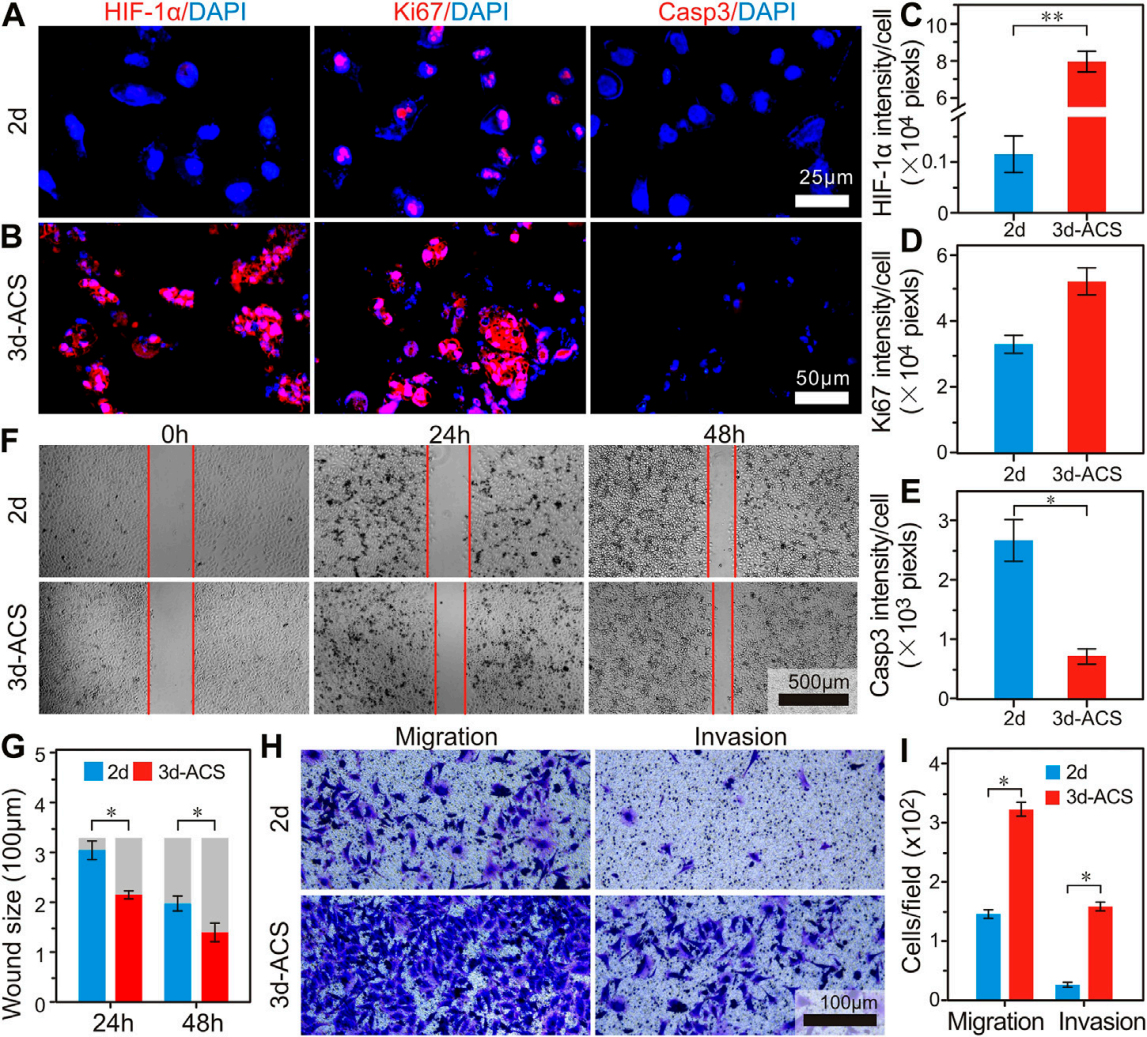
The biological characteristics changes of the GC cells in 3d-ACS and traditional 2d culture. **(A,B)** Immunofluorescence staining of HIF-1α, Ki67, and Caspase3 in MGC-803 cells cultured in 2d petri dish and 3d-ACS. **(C–E)** Quantitative analysis of mean fluorescence intensities of HIF-1α, Ki67, and Caspase3 in 2d culture and 3d-ACS. **(F)** Wound healing experiments of monolayer MGC-803 cells released from 3d-ACS and 2d culture. **(G)** Quantitative results of wound healing experiments. **(H)** The transwell assay to evaluate the ability of migration and invasion of MGC-803 cells released from 3d-ACS and 2d culture. **(I)** Quantitative results of migration and invasion. *n* = 3 per group, *: *p* < 0.05, **: *p* < 0.01.

In 3d-ACS, the GC cells grew like organoids and squeezed each other. The additional alginate shell provided an effective barrier for oxygen diffusion. Using the relationship between [Ru (dpp)_3_]^2+^ Cl_2_ and oxygen concentration (Figure 2E), a typical negative correlation curve was established (Figure 2F). After 7 days of culture, a unique hypoxic environment was well established in the 3d-ACS with an oxygen content of ∼1.29%, whereas the cells cultured in 3d-AC (without shell, ∼16.34%) and Matrigel (∼19.11%) did not achieve comparable results (Figure 2G). In order to detect the induced hypoxia in 3d-ACS, the 2d traditional cultured GC cells were introduced as the control. After 11 days of culture, the 3d-ACS was fixed, sectioned, and HIF-1α immunofluorescence staining was performed. The results manifested that the expression of HIF-1α was significantly upregulated in the nucleus of MGC-803 cells cultured in 3d-ACS (*p* < 0.01), indicating the hypoxia model had been established successfully in 3d-ACS (Figures 3A–C). Additionally, the mean expression of Ki67 in MGC-803 cells indicated that the proliferation of MGC-803 cells in 3d-ACS was similar to that in 2d culture (*p* > 0.05, Figure 3D). However, in Caspase-3, the apoptosis-related proteins expressed in GC cells in 3d-ACS were greatly reduced than that in 2d culture (4.15 folds, *p* < 0.05, Figure 3E), which was consistent with the real situation of cancer cells *in vivo* (Imamura et al., 2015).

### 3.4 The cytological performances of GC cells in 3d-ACS

In order to further evaluate the invasion and metastasis of MGC-803 cells in 3d-ACS, the cell clusters were released from the 3d-ACS and recultured in 2d petri dishes, and the wound healing experiments were performed. Notably, the 3d-ACS culture significantly altered cell motility and time-dependent migration. After 24 h culture, 7.9% of wound healing was achieved in 2d culture, while 34.9% of wound healing was obtained in 3d-ACS. After being cultured for 48 h, the healing rates for 2d culture and 3d-ACS were 40.4% and 57.7% respectively (Figures 3F,G). These findings suggested that the strong malignant phenotype of GC cells *in vivo* had been recovered partially in 3d-ACS. Similarly, migration and invasion tests also demonstrated that the mobility increased 2.2 times of MGC-803 cells in 3d-ACS, and invasiveness elevated to 5.7 times (Figures 3H, I).

### 3.5 Drug sensitivity in 2d and 3d-ACS

**FIGURE 4:**
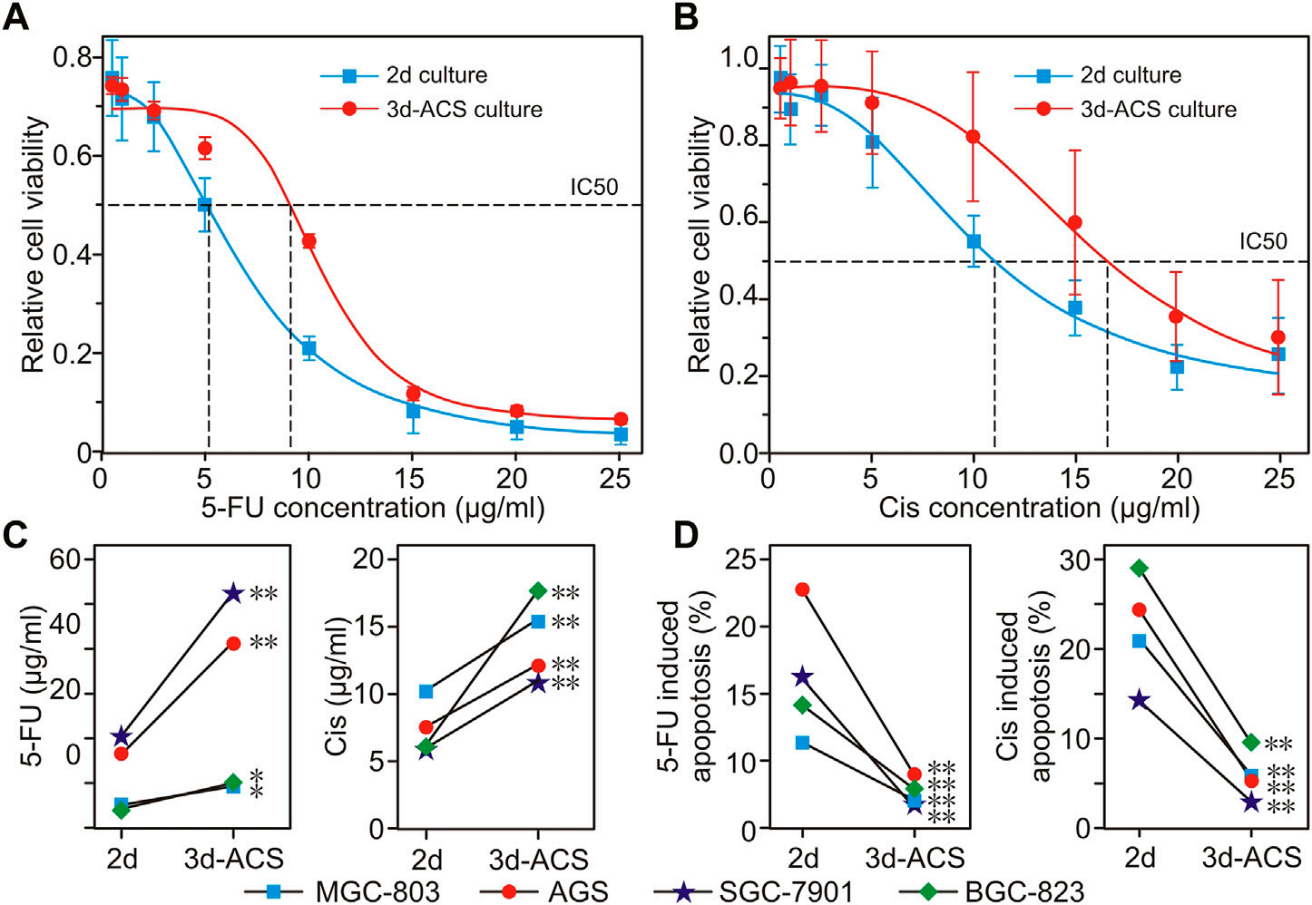
The drug responses of GC cells to 5-FU and cisplatin cultured in normoxia of 2d dish and 3d-ACS. **(A,B)** The reactive changes of MGC-803 cells to 5-FU and cisplatin that cultured in 3d-ACS and 2d dish for 48 h. **(C)** In 2d or 3d-ACS conditions, for different GC cell lines, the IC50 variations of GC cell to 5-FU and cisplatin. **(D)** Under different conditions (2d or 3d-ACS), the GC cell apoptosis is induced by 5-FU or cisplatin. *n* = 3 per group, *: *p* < 0.05, **: *p* < 0.01.

According to the latest guideline of the Chinese Society of Clinical Oncology (CSCO), 5-FU and cisplatin (Cis) were chosen to perform the drug sensitivity tests. The results showed that for 5-FU in 3d-ACS cultured MGC-803 cells, the IC50 increased by 1.76 times compared to that in the 2d dish (5.19 μg/mL vs. 9.11 μg/mL, Figure 4A), while it elevated by 1.41-fold for Cis (11.69 μg/mL vs. 16.51 μg/mL, Figure 4B).

For comparison, except for the MGC-803 cell line, chemotherapy responses were analyzed in several classical GC cell lines of 2d and 3d-ACS culture (i.e., AGS, SGC-7901, and BGC-823). For these cell lines, the organoid-like cell clusters in 3d-ACS manifested similar chemotherapeutic responses compared with that of GC organoids (Li et al., 2019; Lin et al., 2019), and overall higher IC50 values, more resistance, and less apoptosis were obtained in 3d-ACS (Figures 4C, D). It can be deduced that the 3d-ACS greatly enhanced the resistances of these cell lines to 5-FU and Cis, which also suggests possible failures in clinical chemotherapy *in vivo*. In short, the 3d-ACS culture was much closer to the real situation *in vivo*, and inseparable from the formation of a hypoxic microenvironment.

### 3.6 3d-ACS induced variation of gene transcription and proteins expression of GC cells

**FIGURE 5:**
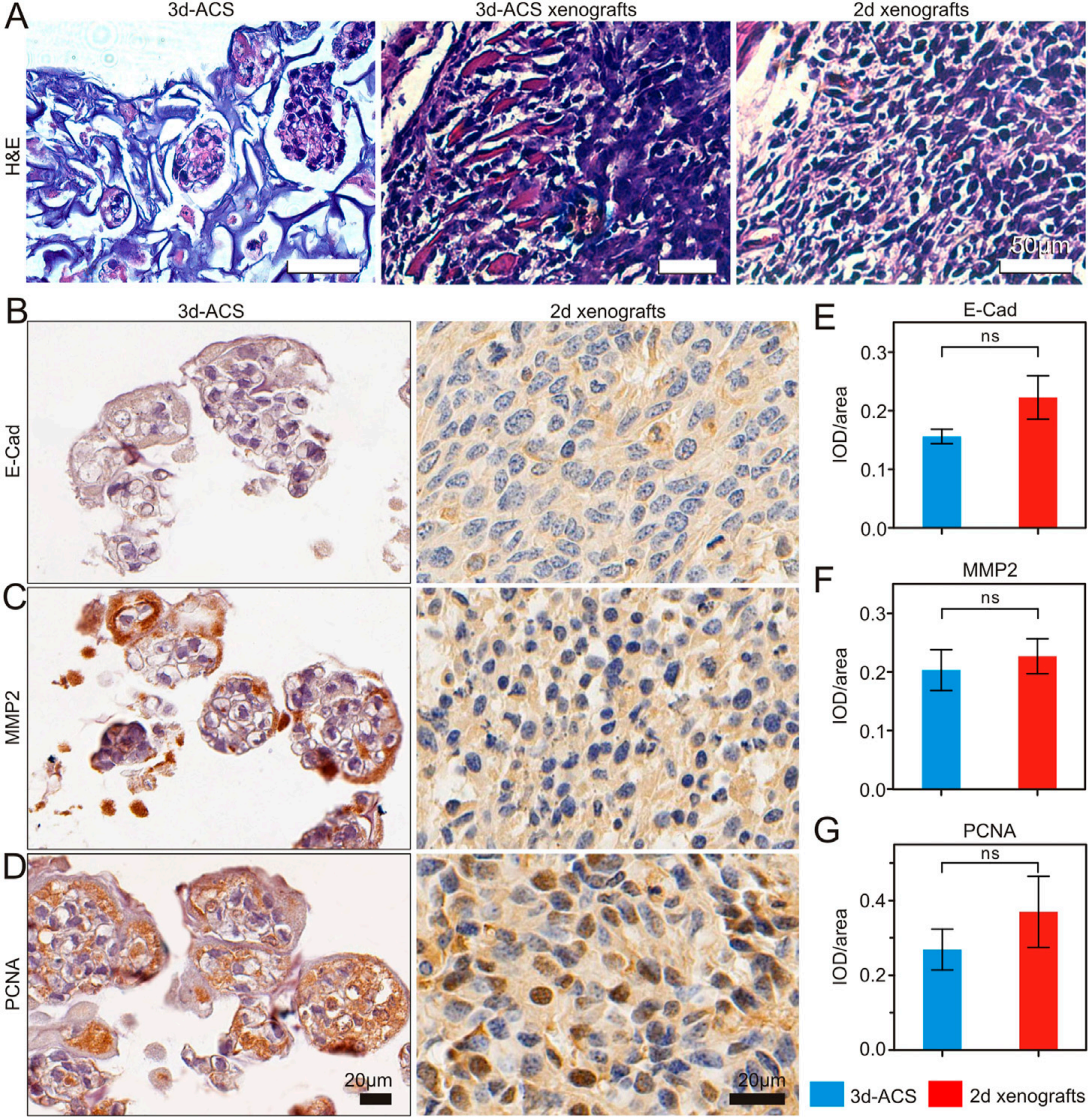
The representative H&E and IHC staining of GC cell line (MGC-803) cultured in 3d-ACS and xenograft tumor tissue. **(A)** Morphology comparison of MGC-803 cells culture 3d-ACS and 3d-ACS or 2d xenograft tumor model. **(B–D)** E-Cad, MMP2, and PCNA IHC staining of 3d-ACS and 2d xenografts tumor tissues. **(E–G)** The expression scores in E-Cad, MMP2, and PCNA (*n* = 3 per group).

Compared with the xenograft tumor tissue established with 2d culture, the cell morphology was very similar to that of conventional (2d) tumor xenografts (Figure 5A). The IHC staining of E-Cad, MMP2, and PCNA revealed that in 3d-ACS, the epithelial-mesenchymal transition (EMT), migration, anti-apoptosis, and the malignant phenotype of GC cells were similar to that of 2d xenografts *in vivo* (Figures 5B–G). It also suggested that a functional hypoxia microenvironment had been established in 3d-ACS *in vitro*, which endowed GC cells with similar malignant phenotypes *in vivo*.

**FIGURE 6:**
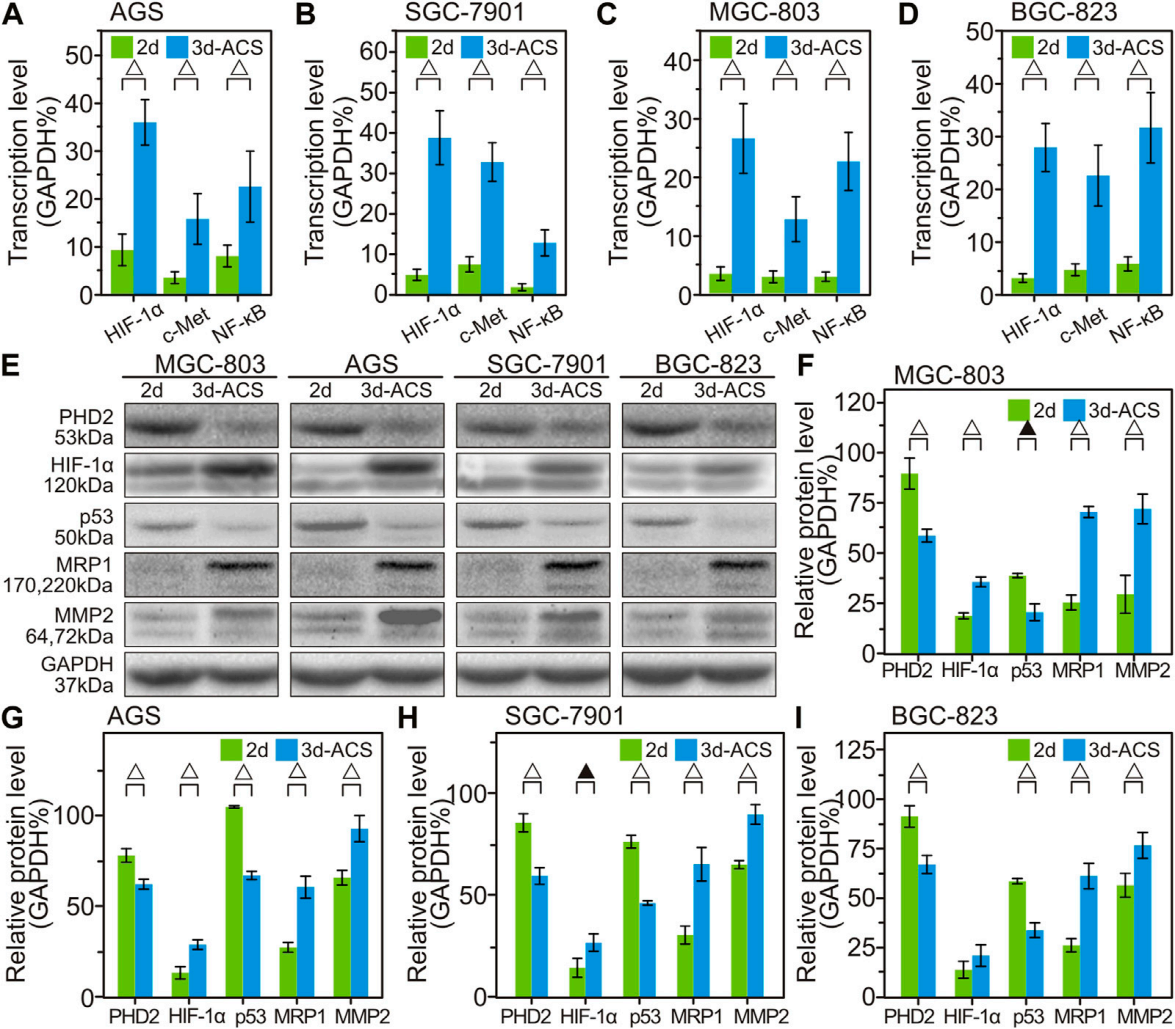
The variation of gene transcription and protein expression in 3d-ACS and 2d culture. **(A–D)** Quantitative analysis of the qPCR data of HIF-1α gene expression (percent of GAPDH) and related changes to cell proliferation, metastasis, and drug resistance in several GC cell lines within 3d-ACS and 2d culture. **(E)** Western blot results of GC cell lines. **(F–I)** Quantitative analysis of the Western blot experiments of MGC-803, AGS, SGC-7901, and BGC-823, respectively. *n* = 3 per group, ▲: *p* < 0.05, △: *p* < 0.01.

For several classical GC cell lines cultured in 3d-ACS, the amplification of hypoxia-related gene showed that the HIF-1α was significantly upregulated, which also resulted in the irregular activation of the c-Met signaling pathway, as well as enhanced tumor formation, aggressive growth, and metastasis (Figures 6A–D). At the same time, in 3 days-ACS, the expression of NF-κB, a transcriptional regulator, was also significantly increased compared with that of 2d cultured GC cells (Figures 6A–D). It suggested that a series of the malignant phenotype of GC cells in 3d-ACS had been activated *via* the classical HIF-1α and NF-κB pathways.

Compared with the GC cells cultured in 2d dishes, the Western blot experiments revealed that the PHD2, the upstream wrecker of HIF-1α, was also significantly decreased (Figures 6E–I). Since the hypoxia often downregulates the hydroxylase activity of PHD2 to prevent HIF-1α degradation, leading to increased HIF-1α protein levels (Chen et al., 2019), it can be convinced that the hypoxia microenvironment had been successfully established in the 3d-ACS. In addition, coinciding with the previous report (Guan et al., 2021), in hypoxia of 3d-ACS, the expression of p53 was reduced due to the inhibitory effects of HIF-1α, thereby inhibiting GC cell apoptosis in 3d-ACS. Moreover, the increased expressions of MRP1 and MMP2 were also observed in 3d-ACS, which suggested the hypoxia 3d-ACS environment caused a wider range of chemotherapeutic resistance and strengthened the metastasis ability of the GC cells.

### 3.7 3d-ACS induced malignancy *in vivo*

**FIGURE 7:**
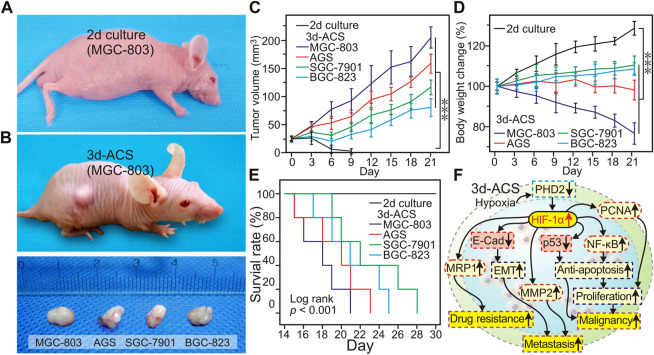
After inoculation in mice, the GC cell growth in the mouse model. **(A)** On day 21, with the same number of inoculation, the tumor morphology of MGC-803 cells cultured in 2d and 3d-ACS, the cells cultured in 2d failed to establish tumor xenografts (up panel). **(B)** After cultured in 3d-ACS, the *ex-vivo* tumor morphology of 4 GC cell lines in mice on the day 21. **(C)** The body weight changes of mice with different cell line inoculation. **(D)** Tumor growth curve of 3d-ACS xenografts (*n* = 5 for each 3d-ACS line). **(E)** Kaplan-Meier curves of mice in different cell line inoculation. **(F)** The mechanisms of 3d-ACS hypoxia-induced malignancy of cancer cells. *n* = 5 per group, ***: *p* < 0.001.

In view of the variation of gene and protein expression, an equal number of GC cells cultured in 2d dish and 3d-ACS were subcutaneously transplanted into mice for tumorigenicity evaluation. As shown in Figures 7A, B, after 1.0 × 10^5^ cells inoculation, no obvious tumors were formed of the GC cells cultured in 2d dishes to the end of the experiment, while the GC cells cultured in 3d-ACS were all formed tumors with a diameter of about 0.6 cm after 3 weeks of inoculation (Figures 7B, C). Mouse body weight (3d-ACS of MGC-803 and AGS group) showed a significant decline with time elapsed (Figure 7D). On the contrary, the body weight showed a steady increase for the mice inoculated with the 2d cultured GC cells (Figure 7D). Additionally, the Kaplan-Meier curve clearly revealed that the malignancy of the GC cell lines had been significantly enhanced due to the education in the hypoxia microenvironment of 3d-ACS (*p* < 0.001), and the survival rate of mice decreased to varying degrees (Figure 7E).

## 4 Discussion

In this study, the alginate and non-toxic calcium ions were introduced to construct a 3 dd microsphere (core) for GC cell culture *in vitro*. In the hydrogel core, the first 7 days of culture effectively imitated the physiological state of newly formed tumor lesions *in vivo*, including sufficient oxygen supply, and excessive cell proliferation, without necrosis inside of the lesion. Using alginate solution with different concentrations, an alginate-based core-shell structure (3d-ACS) was prepared *in vitro*, which restored certain physical and biological properties of tumor tissue *in vivo*, such as stiffness, simulated the hypoxia conditions, as well as the barrier for nutrient and metabolite diffusion. The shell structure of the 3d-ACS permitted water diffusion into the matrix (Zhang et al., 2013), but prohibited the high-speed diffusion of oxygen, which led to hypoxia in the cells mass in the 3d-ACS, and greatly affected the cell respiration and growth (Chandrasekaran et al., 2006).

In the space of 3d-ACS, with the cell growth, interaction, and squeezing of each other, the cell clusters became larger and denser, finally forming organoid-like structures. At the same time, due to the diffusion barrier of the core-shell structure, it made the uneven distribution of oxygen, pH, metabolite, and nutrient, and formed a concentration gradient from the core to the periphery of the 3d-ACS. In TME model design *in vitro*, the previous studies major focused on the dominant parameters such as the stiffness, biochemical ligand, as well as the mutual interactions of different cells in TME (Singh et al., 2018). We proposed that some additional parameters should be detailed in the TME design, which includes hypoxia, pH, and other parameters that may potentially affect the genomic and epigenetic evolutions of GC. 3d-ACS highly recovered the restriction of cell growth and hypoxia in old lesions *in vivo*. Therefore, the 3d-ACS can simulate the hypoxia of the TME *in vivo* and makes it convenient to establish a hypoxia model *in vitro*.

In previous studies, the close relationship had been investigated between hypoxia and the aggressiveness and poor prognosis of GC, as well as the over-expression of HIF-1α in GC of TME (Xia et al., 2020; Li et al., 2021). However, only few studies focused on the hypoxia model and relative mechanisms of GC in the 3d hydrogel model independent of the professional facilities *in vitro*. Compared with the traditional 2d culture in a petri dish, the expression of HIF-1α in GC cells increased significantly in 3d-ACS, and was accompanied by increased Ki67 and decreased caspase3. In addition, the migration and invasiveness of the GC cells were enhanced greatly in 3d-ACS. In the 3d-ACS group, the tumor models were all established with extremely less cells, and distal organ metastases were observed in some mice (MGC-803, 1/5 lung metastasis, BGC-823, 1/5 kidney metastasis, Supplementary Figure S2A, B), as well as the poor prognosis (Figure 7E). Our 3d-ACS model provided a simple preparation, easy repeat, and economic way to conduct the hypoxia study in most laboratories with moderate configuration, without strict requirements of professional anaerobic facilities.

Compared with the CG cells cultured in a 2d petri dish, it found that the GC cells cultured in 3d-ACS were less responsive to the first-line chemotherapeutics (5-FU, cisplatin), and the classical multidrug resistance-associated proteins (MRP1) was also significantly increased (Figure 7F). Therefore, we speculated that it was these discrepancies between 2d culture and 3d-ACS that caused the low translation rate in drug R&D developments (less than 10%) (Cox et al., 2015). Recently, a few studies pointed out that for many anti-cancer trials, the drugs may attack the TME rather than kill the cancer cells, thus exerting anti-cancer effects indirectly (Stamati et al., 2020). These cutting-edge views pushed it forward to use the 3d-ACS model for TME research. To reveal the fundamental mechanisms in the hypoxia model, further study is still needed, while our 3d-ACS model has laid a certain foundation for the biological investigation of GC hypoxia, and it can serve the future test of therapeutic interventions in a defined genetic background for each patient. However, it should be kept in mind that the 3d-ACS consisted only of the GC cell line, without epithelial and mesenchyme layer surrounding, and lack of blood vessels or immune cells. Drugs that target the TME thus cannot be evaluated, while it can be used to analyze the individual interference in a defined setting.

Overall, our 3d-ACS model has overcome many current limitations of 2d culture to establish and/or simulate the “real hypoxia” TME *in vitro*, without the need for any professional equipment. In this model, mutual interactions, and the gradient distributions of nutrients and oxygen can be reproduced much closer to the TME *in vivo*. In addition, the 3d-ACS provides a new way for the discoveries of chemotherapeutics and preclinical drug screening, as well as more reliable information on pharmacological research *in vitro*.

## 5 Conclusion

In summary, the alginate-based core-shell structure (3d-ACS) had been successfully generated for human GC cell culture *in vitro*. In the 3d-ACS, the entrapped GC cells survived prosperously, and multiple organoid-like cell clusters were established. The 3d-ACS structure closely mimicked the hypoxia TME of GC *in vivo*, promoted the cell malignant phenotype, impacted the drug responses, and enhanced the cell malignancy in animal models. Compared with the traditional 2d culture, the 3d-ACS provided the possibility to get closer to the real TME *in vivo*, shortened the discrepancies between traditional 2d cell-line-based drug screening and clinical trials, as well as the potential for personalized anticancer strategy development.

## Data Availability

The raw data supporting the conclusion of this article will be made available by the authors, without undue reservation.
