# Supplementary material for: Three-dimensional core-shell alginate microsphere for cancer hypoxia simulation in vitro
Source: Front Bioeng Biotechnol. 2023 Apr 11;11:1174206. doi: 10.3389/fbioe.2023.1174206 (PMC10126516; doi:10.3389/fbioe.2023.1174206)
Supplement: Supplementary file 1 [file DataSheet1.doc]

**Appendix A. Supplementary material**

# Three-dimensional core-shell alginate microsphere for cancer hypoxia simulation *in vitro*

Yejiao Ruan^1†^, Lingyun He^1†^, Jiamin Chen^1^, Jinfeng Wang^1^, Shujing Zhao^1^, Xiaoling Guo^1^, Yao Xie^3^, Zhenzhai Cai^1*^, Xian Shen^2*^, Chao Li^1*^

^1^The Second Affiliated Hospital and Yuying Children's Hospital of Wenzhou Medical University, Wenzhou, China.

^2^The First Affiliated Hospital of Wenzhou Medical University, Wenzhou, China.

^3^Beijing Automation Control Equipment Institute, Beijing, China.

†Yejiao Ruan and Lingyun He contributed equally to this work and share the first authorship.

*** Correspondence:**

Zhenzhai Cai, caizhenzhai@wmu.edu.cn, Xian Shen, shenxian@wmu.edu.cn, Chao Li, dishboy@163.com

**S1. Immunohistochemistry (IHC) and immunofluorescence staining of 3d-ACS**

The 3d-ACS sections were firstly incubated with primary antibody (E-Cad, MMP2, PCNA, listed in Table S1) at 4°C overnight. After wash, the sections were incubated with the HRP or fluorophores-labeled secondary antibody (Table S1). For IHC, the slides were colored with DAB (Dako, Cytomation, CA, USA) and counterstained with hematoxylin. The slides were mounted with resin and observed under microscopy (Leica DM2500) as routine.


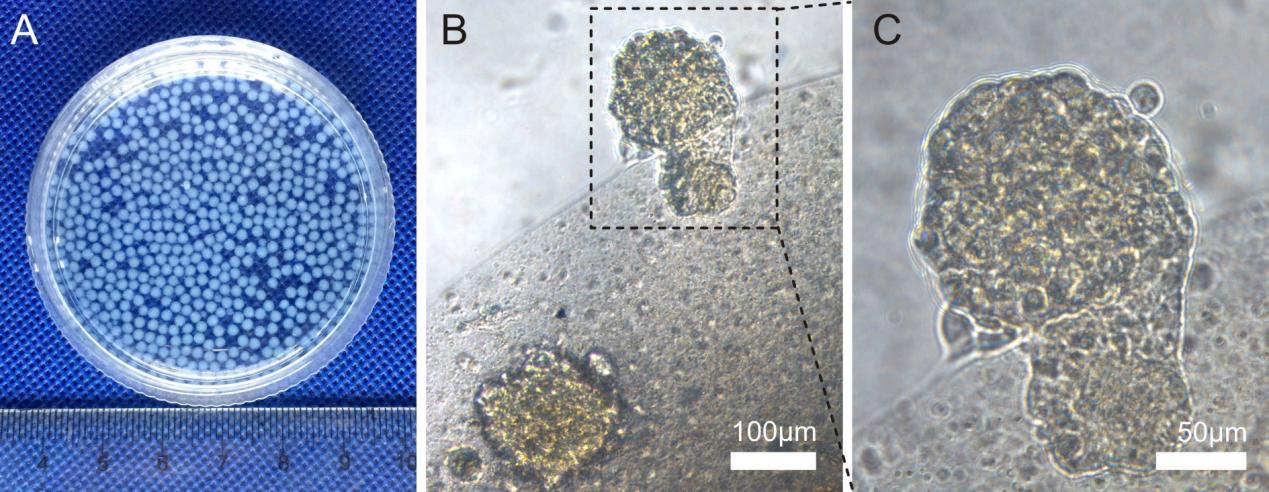


**Fig. S1.** The morphologies of fabricated 3d-ACS and the tumor cell growth in it. (A) The photo of the fabricated 3d-ACS. (B) The cell growth in 3d-ACS edge and the clone squeezed out from the cavity. (C) Partial enlargement of the metastasis-like structure.

**
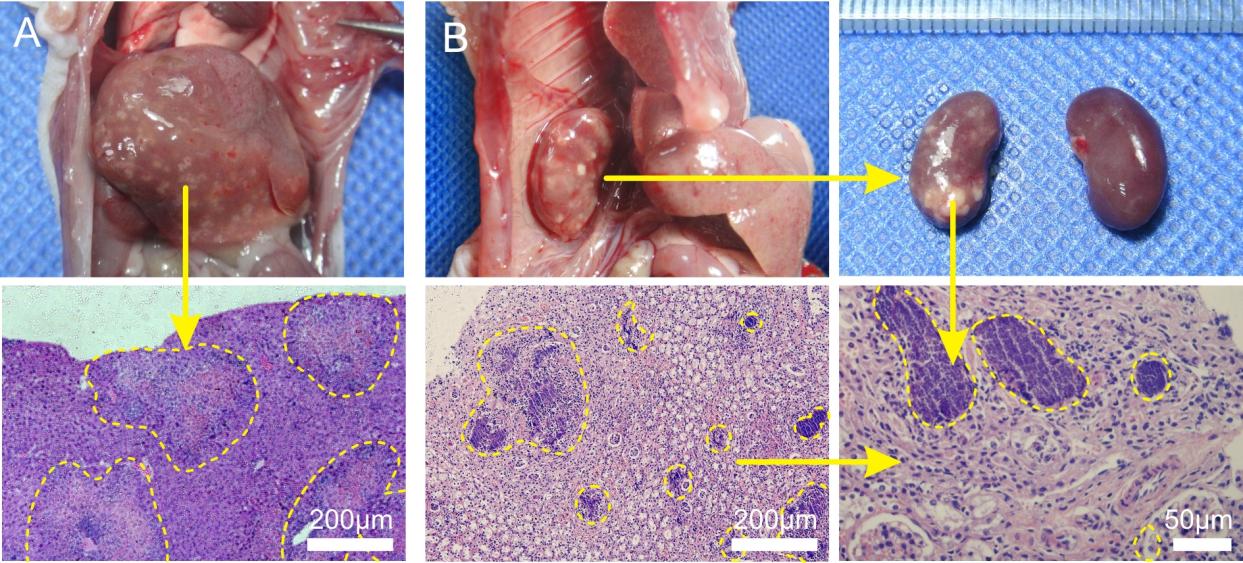
**

**Fig. S2.** The distal organ metastases in mice transplanted with GC cells cultured in 3d-ACS. (A) The liver metastasis of MGC-803 cells and the tissue section with H&E staining. (B) The kidney metastasis of BGC-823 and the tissue section with H&E staining and different magnifications.

Table S1. The antibody list used in this research.

| **Primary antibody** | **Vendor** | **ID** | **Product Name** |
| --- | --- | --- | --- |
| Ki67 | Abcam | ab15580 | Rabbit polyclonal to Ki67 |
| Caspase-3 | Abcam | ab32351 | Rabbit monoclonal [E87] to Caspase-3 |
| HIF-1α | Abcam | H1alpha67 | Mouse monoclonal [H1alpha67] to HIF-1 alpha |
| E-Cadherin | Abcam | EP700Y | Rabbit monoclonal [EP700Y] to E Cadherin - Intercellular Junction Marker |
| MMP2 | Abcam | ab86607 | Mouse monoclonal [6E3F8] to MMP2 |
| PCNA | Abcam | ab92552 | Rabbit monoclonal [EPR3821] to PCNA |
| PHD2 | Abcam | ab226890 | Rabbit polyclonal to PHD2 / prolyl hydroxylase - C-terminal |
| p53 | Abcam | ab26 | Mouse monoclonal [PAb 240] to p53 |
| MRP1 | Abcam | ab233383 | Rabbit monoclonal [EPR21062] to MRP1 |
| GAPDH | Abcam | ab9485 | Rabbit polyclonal to GAPDH |
| **Secondary antibody** | Abcam | ab96879 | Goat Anti-Mouse IgG H&L (DyLight® 488) preadsorbed |
|  | Abcam | ab150083 | Goat Anti-Rabbit IgG H&L (Alexa Fluor® 647) preadsorbed |
|  | Abcam | ab150077 | Goat Anti-Rabbit IgG H&L (Alexa Fluor® 488) |
|  | Abcam | ab205719 | Goat Anti-Mouse IgG H&L (HRP) |
|  | Abcam | ab205718 | Goat Anti-Rabbit IgG H&L (HRP) |

**Table S2.** The primers list of for RT-PCR amplification and the references.

| **Primer** | **5’-3’ sequence** | **Product length (bp)** | **Ref.** |
| --- | --- | --- | --- |
| HIF-1α (F) | GAACGTCGAAAAGAAAAGTCTCG | 124 | (Zhao et al. 2019) |
| HIF-1α (R) | CCTTATCAAGATGCGAACTCACA |
| c-Met (F) | AACTGGTGTCCCGGATATCA | 276 | (Uen et al. 2006) |
| c-Met (R) | ATATTCTTTGCTCCTTGCCA |
| NF-κB (F) | GAAATTGGCCAAGGTCTGATA | 155 | (Chen et al. 2015) |
| NF-κB (R) | TCGTAGAATCCCCGCACA |
| GAPDH (F) | CCTCAAGATCATCAGCAATGC | 165 | (Uen et al. 2006) |
| GAPDH (R) | GGAAACTGTGGCGTGATGG |

**References**

Chen L, Han Y, Chen Y, Li Z, Wang H, Liu Y, et al. (2015). Eight SNVs in NF-κB pathway genes and their different performances between subclinical mastitis and mixed Chinese Holstein cows. Gene. 555:242-249. doi:10.1016/j.gene.2014.11.011

Uen Y-H, Lin S-R, Wu C-H, Hsieh J-S, Lu C-Y, Yu F-J, et al. (2006). Clinical significance of MUC1 and c-Met RT-PCR detection of circulating tumor cells in patients with gastric carcinoma. Clin Chim Acta. 367:55-61. doi:10.1016/j.cca.2005.11.013

Zhao L, Ma R, Zhang L, Yuan X, Wu J, He L, et al. (2019). Inhibition of HIF-1a-mediated TLR4 activation decreases apoptosis and promotes angiogenesis of placental microvascular endothelial cells during severe pre-eclampsia pathogenesis. Placenta. 83:8-16. doi:10.1016/j.placenta.2019.06.375
